# Long-Term Tissue Preservation at Ambient Temperature for Post-Mass Fatality Incident DNA-Based Victim Identification

**DOI:** 10.3390/genes15030373

**Published:** 2024-03-19

**Authors:** Xavier Liang Shun Chan, Shumei Michelle Lai, Danial Asyraaf bin Hamdan, Yee Bin Ng, Onn Siong Yim, Christopher Kiu Choong Syn

**Affiliations:** DNA Profiling Laboratory, Biology Division, Health Sciences Authority, 11 Outram Road, Singapore 169078, Singapore

**Keywords:** freezing, salt, alcohol, chemical preservative

## Abstract

In a mass fatality incident (MFI), effective preservation of tissue samples is the cornerstone for downstream DNA-based identification of victims. This is commonly achieved through freezing of tissue samples excised from bodies/fragmented remains which may be buried or stored in refrigerated containers. This may, however, not be possible depending on the nature of the MFI; in particular, during armed conflict/war where extended periods of electrical outages would be expected. The present study compared the effectiveness of long-term tissue preservation at ambient temperatures using two commercial products (non-iodized kitchen salt and a 40% alcoholic beverage) against a chemical preservative (Allprotect™ Tissue Reagent (Qiagen, Germantown, MD, USA)) and freezing at −20 °C. Bovine muscle tissue, used as a proxy for human tissue, was treated with the four preservation methods and sampled at six different time-points over a 24-month period. All four methods were able to preserve the bovine tissue, generally yielding STR-PCR (Short Tandem Repeat-Polymerase Chain Reaction) amplicons > 200 bp in size even at the end of 24 months. Gel electrophoresis, however, indicated that salt was more effective in preserving DNA integrity with high-molecular-weight DNA clearly visible as compared to the low-molecular-weight DNA smears observed in the other methods. This study also proposes a simple process for the rapid and low-cost preservation of tissue samples for long-term storage at ambient temperatures in support of post-incident victim identification efforts.

## 1. Introduction

The identification of victims from civil (e.g., natural disasters) or national emergencies (e.g., war) is typically achieved through one of three primary modes, namely Friction Ridge analysis, Odontology or DNA analysis [1]. Of these three, the DNA result is the only primary identifier that allows for identification through comparison with non-self information (i.e., the DNA results of biological relatives). It may also be the best method for re-associating highly fragmented remains, especially if they are scattered across large distances, for example, in a plane crash incident. Efficient DNA analysis via standard STR-PCR-based methods is, however, dependent on the remains being reasonably well preserved. While decomposition in cold weather (5 to 13 °C) is slow due to the limited growth of insects (maggots), the same cannot be said for the warm and humid conditions encountered in the tropics, where complete skeletonization may occur within 4 weeks [2]. Indeed, the rapid decomposition of remains was a major challenge to the recovery of usable DNA for analysis after the Indian Ocean Earthquake and Tsunami of 2004, especially in the immediate aftermath of the incident due to the lack of chilled storage facilities [3]. As such, effective collection and preservation of tissue samples from recently deceased remains is a critical step for successful post-incident DNA-based identification and body parts re-association.

The previous literature has reported successful tissue preservation (up to 1 year) using a readily available household chemical: kitchen salt [4,5,6,7,8,9]. Kitchen salt is easily available and usually affordable in most parts of the world. Its hygroscopic nature retards bacterial and fungal growth while preventing the hydrolysis of DNA molecules without the use of chemical preservatives, which may be costly and/or difficult to procure when required during the incident. More importantly, salt has been used throughout history, reportedly as early as 2000 BCE in Egypt, as a means to preserve food in the absence of refrigeration [10]. Ethanol is another chemical that is known to be germicidal and has a preservative effect on animal tissue [5,8,11]. It has been readily available in the form of drinking alcohol since antiquity in most human cultures and societies and has been used as an impromptu microbicide agent in emergencies [12,13]. For example, alcohol in the form of drinking alcohol and as a disinfectant can be easily acquired from supermarkets/grocery stores in Singapore. Other studies have also been performed on the use of drinking liquors on tissue preservation [4,14]. Such readily available chemicals may be of particular relevance in terms of preserving soft tissue samples of victims for subsequent DNA-based identification in national emergencies where there may be prolonged disruption to electrical power supply.

This report presents our observations on the use of kitchen salt and drinking alcohol to preserve soft tissue samples for up to 24 months at ambient temperatures for forensic identification purposes. These two preservation methods were compared against two other established methods—chemical preservatives and freezing at −20 °C. Due to sample limitations and bioethics considerations, bovine tissue samples were used as proxies for human tissue samples in this study.

## 2. Materials and Methods

### 2.1. Experimental Design

Four tissue preservation methods were compared for this study:

1. Non-iodized kitchen salt (Pagoda, Beijing, China) at ambient temperature (∼25 °C);
2. Allprotect™ Tissue Reagent (Qiagen, Germantown, MD, USA) at ambient temperature;
3. Vodka, 40% ethanol by volume (Absolut, Stockholm, Sweden) at ambient temperature;
4. Freezing at −20 °C without cryoprotectants.

Steak-cuts from two different cows were purchased from a local butcher. A total of 0.3 g of samples were prepared from each cut and preserved in (1) 15 g of kitchen salt; (2) 5 mL of Allprotect™ Tissue Reagent; (3) 5 mL of vodka; and (4) in a freezer at −20°. Bovine samples preserved in salt were stored using resealable polyethylene bags measuring about 5 cm by 4 cm, while the samples preserved in liquid preservation mediums and in a freezer were stored in Axygen^®^ 5 mL screw-cap polypropylene tubes (Corning Inc., Corning, NY, USA).

The bovine samples were kept at corresponding temperatures over the entire study period of 24 months. The effects of the preservation methods on the DNA were investigated across 6 time-points: 7 days, 1 month, 4 months, 12 months, 16 months, and 24 months. It should be noted that the manufacturer-recommended maximum duration of ambient temperature tissue storage in Allprotect™ Tissue Reagent is 7 days, while storage beyond 12 months would require the samples to be frozen. However, for this study, the samples in Allprotect™ Tissue Reagent were kept at ambient temperatures to eliminate the effects of freezing.

### 2.2. DNA Processing and Comparisons

Three 0.3 g pieces of tissue from each cow were retrieved from the fresh steak cut (day 0; fresh control) and from each time-point for each preservation method for DNA processing, yielding a total of six samples per time-point per method. The tissue samples, except for the fresh control samples and samples preserved in the freezer, were rinsed briefly in ultrapure water to remove the preservation chemicals. DNA extraction was performed using a DNA IQ™ Casework Extraction Kit and DNA IQ™ Casework Pro Kit on a Maxwell^®^ FSC instrument (Promega, Madison, WI, USA). As the tissue samples preserved in salt, vodka, and Allprotect™ Tissue Reagent had become dehydrated, an increase in extraction buffer (from 400 μL to 700 μL) and lysis buffer (from 200 μL to 350 μL) was applied to all samples to ensure treatment consistency. DNA was eluted in a final volume of 56 μL.

Two µL of each purified DNA sample were assayed using QuantiFluor^®^ ONE dsDNA System (Promega, Madison, WI, USA) on a Quantus™ Fluorometer (Promega, Madison, WI, USA) to determine the double-stranded DNA yield. STR-PCR amplification was performed using a StockMarks^®^ Kit for Cattle—Bovine Genotyping Kit (Applied Biosystems, Foster City, CA, USA) on a VeritiPro™ Thermal Cycler (Thermo Fisher Scientific, Waltham, MA, USA) for 29 cycles with the following cycling conditions: 94 °C for 45 s, 61 °C for 45 s, and 72 °C for 60 s. A 10 min activation at 95 °C before the cycling and a final extension of 72 °C for 1 h were used. The input amount of template DNA was normalized to 1 μL of DNA for the fresh samples and the preserved samples across all time-points instead of the manufacturer’s recommendation of between 1 and 10 ng DNA in a maximum of 1 µL template volume. This approach would better highlight the differences in both quantity (e.g., peak height) and quality (e.g., ski-slopes) of DNA recovered from the well-preserved vs. poorly-preserved samples.

Amplicon separation and detection were performed using a 50 cm capillary on an ABI PRISM^®^ 3500 Genetic Analyzer (Applied Biosystems, Foster City, CA, USA) with an injection parameter of 1 μL at 15 kV 180 s. Generated electropherograms (EPG) were analyzed with GeneMapper^®^ ID-X software v1.6 (Thermo Fisher Scientific, Waltham, MA, USA) with an analytical threshold of 110 RFU. A StockMarks^®^ Kit for Cattle—Bovine Genotyping Kit does not have an allelic ladder; hence, the amplicons are identified based on their base-pair size as stated in the manufacturer’s user guide. The bovine DNA profiles recovered from the preserved samples were compared against the fresh controls.

Gel electrophoresis of the purified DNA from samples at 7 days, 12 months, and 24 months was conducted on an E-Gel™ Power Snap Electrophoresis System (Thermo Fisher Scientific, Waltham, MA, USA) using E-Gel™ EX Double Comb 2% agarose gels (Invitrogen, Carlsbad, CA, USA) to assess the DNA quality. DNA extracted from fresh samples and at 7 days, 12 months, and 24 months of preservation were visualized using gel electrophoreses to visually assess DNA integrity. A total of 50 ng of DNA in a volume of 20 μL were electrophoresed for 6 min using an E-Gel™ EX Double Comb 2% separation protocol alongside an E-Gel™ 1 Kb Plus DNA Ladder (Invitrogen, Carlsbad, CA, USA).

### 2.3. Statistical Analyses

Two variables were evaluated: (i) mean total DNA yield and (ii) percentage of alleles recovered. For statistical data analyses, the triplicate readings from both cows (6 samples in total) for DNA yield were averaged, while the number of alleles observed in the DNA profile of each sample was tabulated as a percentage of alleles recovered. For the percentage allele recovery, it was observed that the DNA profiles from the fresh and 7-day preserved samples had no allele at locus SPS115. Hence, for consistency, locus SPS115 was excluded from all analyses, leaving only 10 loci for each profile. Shapiro–Wilks tests were conducted to evaluate data deviation from a normal distribution and Friedman tests were conducted to compare the DNA yield variables among different preservation methods. Statistical analyses were performed using Python 3 with the SciPy and Scikit packages.

## 3. Results

### 3.1. DNA Recovery and Stability

**Figure 1:**
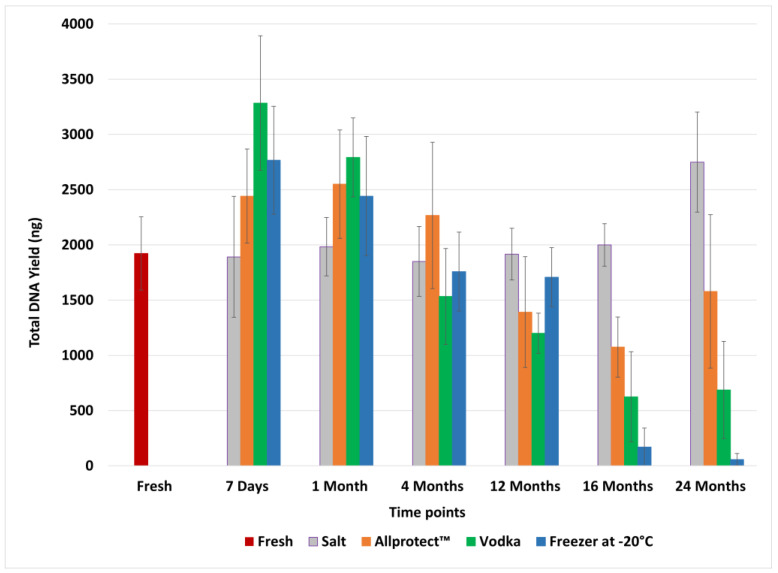
Mean ± SD of total DNA yield of fresh bovine samples and bovine samples preserved in salt, Allprotect™ Tissue Reagent, vodka, and by freezing at −20 °C.

The DNA yield across the six time-points is presented in Figure 1. Mean total DNA yield ranges from 58 ng to 3283 ng, with a declining trend seen over time for all preservation methods except for salt. Fresh, unpreserved samples produced a mean yield of 1923 ng, with a standard deviation (SD) of 332 ng. Table A1 in Appendix A shows the mean total DNA yield with SD in nanograms for each time-point by preservation method, as well as the total number of recovered alleles. Frozen samples recorded the largest decrease in DNA recovery over the 24-month period.

The standard deviations of the DNA yields were observed to be large, even between the triplicate samples from the same cow. To confirm this observation, a separate test to evaluate the variances in DNA yield was performed using another steak cut purchased from the same butcher. Twelve pieces of sample, each 0.3 g, were prepared from this new steak cut and were subjected to the same DNA extraction and quantification procedures in the absence of preservation treatment in order to evaluate this variation in DNA yield. The mean DNA yield was 1529 ng, with an SD of 243 ng. Brown–Forsythe tests were conducted to compare the variance in the new dataset with the variances in DNA yield at each time-point, separated by the method of preservation. The variation observed between the replicate samples preserved in salt, Allprotect™ Tissue Reagent, and vodka was found to be comparable with that of the fresh tissue samples (all *p* > 0.199; F-statistic range: 0.699 to 1.511). Only frozen samples had variances that were significantly different from fresh tissue (F-statistic = 2.931; *p* = 0.018), and this difference is likely an effect of the low DNA recovery from the frozen samples at the later time-points. Very low DNA content will also translate into smaller absolute values of standard deviations, increasing the departure of the variance from previous time-points. These results suggest that the DNA yield variance observed in our preserved samples are likely due to the individual bovine flesh samples. While all the preserved bovine samples were derived from the two steak cuts (3 × 0.3 g from each steak cut), each 0.3 g bovine sample may contain differing proportions of cell types (e.g., muscle cells, fat cells, epimysial connective tissue, etc.), resulting in an inherent DNA yield variance. The full results of the Brown–Forsythe tests are shown in Table A2 in Appendix A.

**Figure 2:**
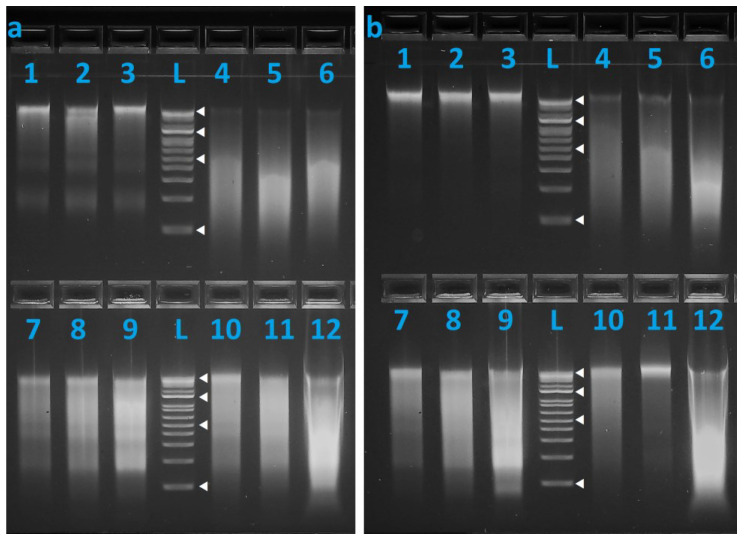
Gel electrophoresis of bovine DNA recovered from (**a**) Cow 1 and (**b**) Cow 2 preserved for 7 days, 12 months, and 24 months. Four preservation methods were used: salt (Lanes 1–3), Allprotect™ Tissue Reagent (Lanes 4–6), vodka (Lanes 7–9), and freezing at −20 °C (Lanes 10–12). Ladder (L) used was 1 kb Plus ladder (15 kb to 100 bp) with white solid triangles (◁) denoting 100, 500, 1500, and 15,000 bp.

**Figure A1:**
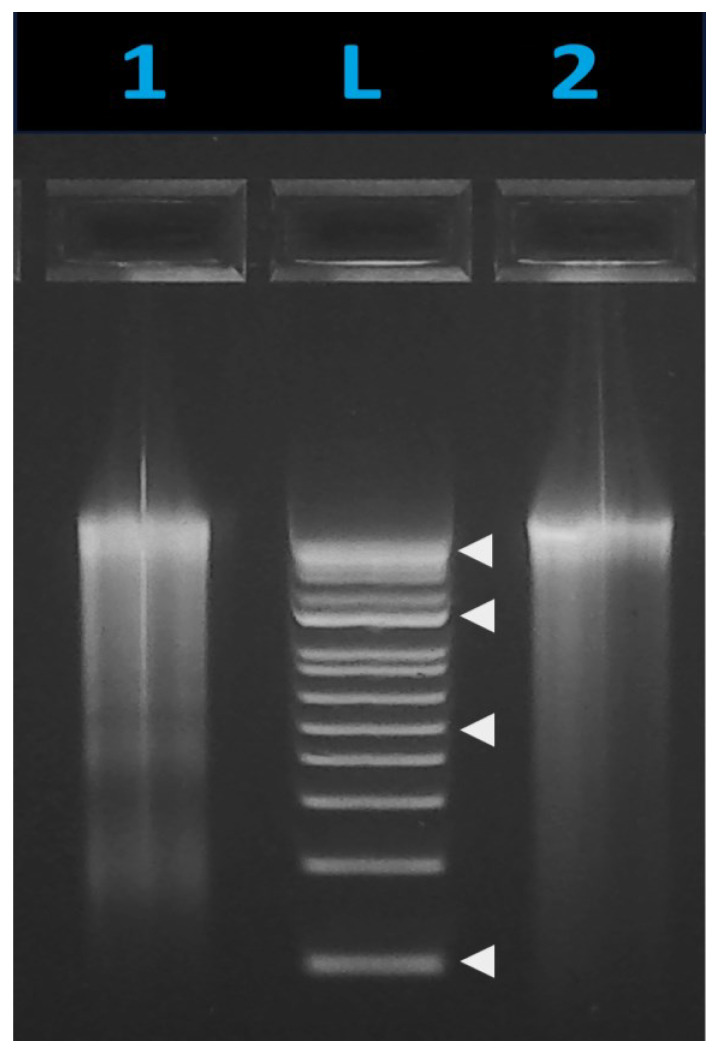
Gel electrophoresis of bovine DNA recovered from Cow 1 (Lane 1) and Cow 2 (Lane 2) at day 0 (fresh control) prior to preservation. Ladder used was 1 kb Plus ladder (15 kb to 100 bp) with white solid triangles (◁) denoting 100, 500, 1500, and 15,000 bp.

DNA quality of the fresh control (Figure A1) and the preserved samples (7 days, 12 months, 24 months) was assessed using agarose gel electrophoresis (Figure 2). Increased smearing indicative of DNA degradation over time could be observed for all preservation methods except for salt, where high-molecular-weight DNA was still clearly visible even at the 24-month time-point. It should be noted that DNA profiles recovered from most samples, less SPS115, were complete. This suggests that although there was DNA degradation, the DNA was sufficiently intact (with respect to the amplicon size range of 65 to 235 bp in the StockMarks^®^ bovine genotyping kit, less locus SPS115) to allow for successful STR-PCR amplification.

### 3.2. Genotyping Allele Recovery

**Figure 3:**
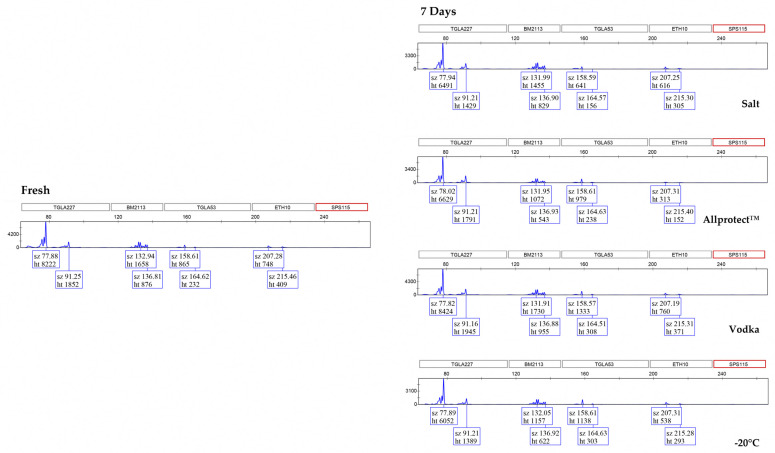

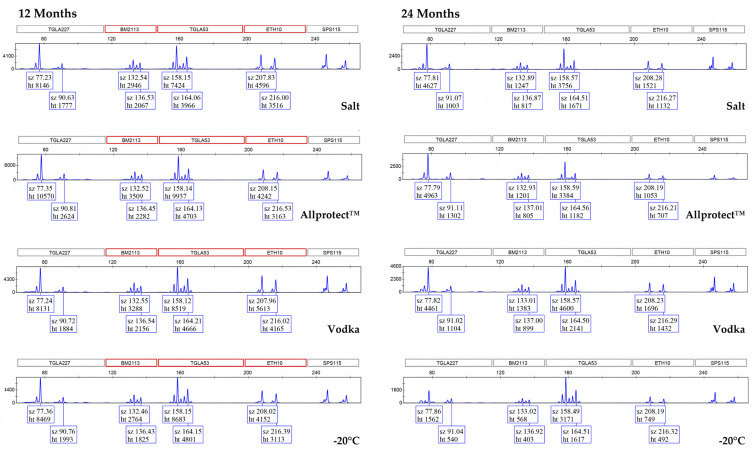
Electropherograms (showing only blue channel) of bovine DNA STR-PCR amplicons generated from fresh samples and samples preserved in salt, Allprotect™ Tissue Reagent, vodka (40% ethanol), and freezing at −20 °C for 7 days, 12 months, and 24 months. All samples yielded the complete DNA profile with the exception of 24-month-frozen bovine samples, which yielded ~97% allele recovery. sz—size, ht—height. Locus SPS115 was excluded from the allele count.

Multiple peak patterns were observed in each locus of the bovine DNA profile (Figure 3). It should be noted that these multiple peaks are not due to mixtures of bovine samples—the same pattern is found in the fresh control samples. Rather, these multiple peaks are a result of the StockMarks^®^ Kit for Cattle—Bovine Genotyping Kit being comprised of di-nucleotide repeats, which inherently have higher levels of stuttering, thereby giving rise to −1, −2, −4, and even −6 repeat stutters [15,16].

By comparing to the fresh control samples, it can be seen from the EPGs that complete bovine DNA profiles (excluding locus SPS115) were recovered from all the preserved samples throughout the 24-month period, with the exception of samples preserved in the freezer at −20 °C for 24 months (97% allele recovery). Interestingly, we observed a noticeable ‘ski-slope’ effect in the fresh and 7-day preserved samples, which resulted in the drop-out of locus SPS115. However, for all the preserved samples at the later time-points, this ‘ski-slope’ effect diminishes, resulting in peaks observed at the locus SPS115 (as seen in the 12-month and 24-month EPGs). For consistency of the results across all time-points, the decision was made to exclude this locus from the subsequent analysis of percent allele recovery. This phenomenon was attributed to PCR inhibition as the quality of the DNA profiles improved after 30 days of preservation. The likely inhibitory agent is the fresh myoglobin in the muscle tissue, which contains Fe^2+^ ions. The half-maximal inhibitory concentration (IC_50_) of Fe^2+^ is 0.59 mM, whereas that of Fe^3+^ is 1.60 mM [17]. As such, PCR inhibition can be expected to be more severe with fresh tissue, resulting in the larger loci having a lower relative fluorescence unit (RFU) and even dropping out as compared with ‘older’ tissue, where the Fe^2+^ would have oxidized over time into Fe^3+^. There is also the additional possibility of Fe^2+^ being drawn out by the preservation mediums over time, which would have reduced the PCR inhibitory effects of Fe^2+^.

### 3.3. Statistical Analyses

The results of the Shapiro–Wilks tests for normality of data distribution indicate that only Allprotect™-preserved tissue yielded DNA quantities that are likely to be from a normal distribution (test statistic = 0.972, *p* = 0.494). The other three preservation methods rejected the null hypothesis that the observed values were from normal distributions (all *p* < 0.046). The full Shapiro–Wilks test results are presented in Table A3 in Appendix A.

The Friedman test statistic and *p*-value for comparison of mean total DNA yield under the four preservative methods are 1.800 and 0.615, respectively. These results fail to reject the null hypothesis that there are no significant differences in the total DNA yield of the four tested methods. Thus, all four methods can be said to have performed comparably in preserving soft tissue for up to 24 months.

## 4. Discussion

DNA analysis is one of the primary methods for the identification of victims from mass fatality incidents. However, achieving a high first-pass rate of obtaining a usable victim’s DNA profile is dependent on the upstream effective preservation of tissue samples. Depending on the scale and the nature of the MFI, sample preservation using refrigerated containers may not be possible due to accessibility and shortage of electrical supply. For example, for MFIs in remote and inaccessible places, the Australian Defense Forces have prepared portable battery-powered refrigerator/freezer units that weigh over 60 kg each when empty for the storage and transportation of tissue samples [18]. Factoring in the requirement for chargers, a reliable source of electricity, and transporting the freezers, it will be challenging to scale up and maintain such a system for weeks or possibly months. This would indeed be the case for armed conflicts such as war, where victims would possibly have been buried in mass graves, and post-incident identification efforts would take place many months or even years after the MFI. Hence, the primary objective of this study is to evaluate the use of readily available mediums that would allow for long-term ambient temperature preservation of tissue samples.

This study evaluated the effectiveness of non-iodized kitchen salt, vodka, and Allprotect™ Tissue Reagent at ambient temperature and freezing at −20 °C for the preservation of soft tissue samples over a prolonged period of 24 months. DNA was successfully recovered from samples preserved using all four preservation methods after 24 months, with the majority of samples yielding complete bovine DNA profiles (excluding locus SPS115). As shown in the gel electrophoresis results where despite signs of DNA degradation in samples preserved in vodka, Allprotect™ Tissue Reagent, and by freezing at −20 °C, the majority of the DNA fragments could be observed to be at the 200 bp to 300 bp region (Figure 2). However, for the samples preserved in salt, most of the DNA recovered were high-molecular-weight fragments localized at the top of the gel.

The effectiveness of salt as a preservation medium reported in our study corroborated the findings of Caputo et al. [7], where one-year salt-preserved human samples yielded DNA that was comparable to that recovered from fresh human samples. Therefore, we do not anticipate any substantial differences in the mechanics of tissue preservation in salt and downstream DNA processing between human or bovine samples. In GlobalFiler™ (Thermo Fisher Scientific, Waltham, MA, USA) and PowerPlex^®^ Fusion 6C PCR STR amplification kits (Promega, Madison, WI, USA), the sizes of the loci range from ~80 bp to 460 bp and ~70 bp to 480 bp, respectively. With the DNA recovered from salt-preserved samples being of high molecular weight, full human STR profiles can be expected even from samples preserved for 24 months at ambient temperatures.

**Figure 4:**
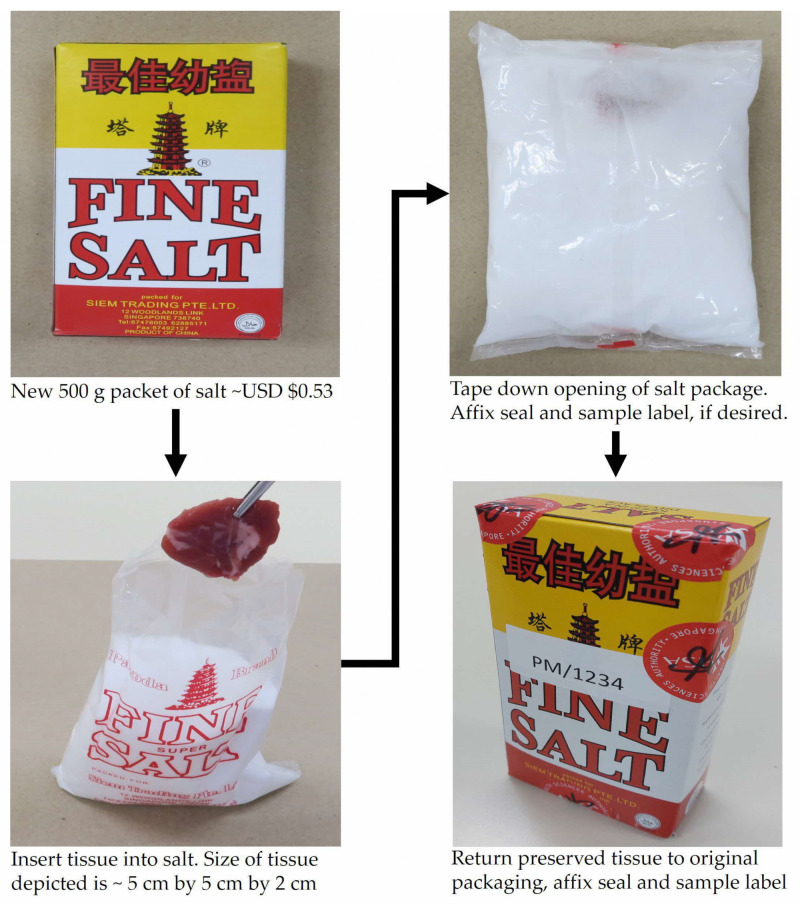
Approach for preservation of samples using a typical package of salt (500 g) from the local supermarkets. The snipped bovine muscle (representing a human tissue sample) is inserted into the salt, which is then returned to its original cardboard box packaging.

We would further highlight the ease by which the salt preservation method can be applied as proposed in Figure 4. In a war scenario, a small body part such as the toe or the buttock flesh of the victim can be snipped for preservation in salt at ambient temperature—this is a relatively minor disfigurement that can be readily concealed with socks/shoes or pants, to protect the dignity of the deceased. Having such a tissue sample collected early would greatly enhance the post-incident identification of victims as opposed to attempting to conduct DNA analysis on remains that would have undergone substantial decomposition or even skeletonization. In the proposed approach, the piece of bovine muscle (representing a toe) can be simply inserted into the salt, and the salt package is then sealed and labeled. Such salt packages would be easy to transport with little to no risk of biological fluid leakage as fluids would be absorbed by the hygroscopic salt. There is also minimal preparatory work involved as there is no need for aliquoting of liquid chemical preservatives, other than the stocking up of salt. Importantly, salt is a very inexpensive material; at the time of this study, a packet of kitchen salt is USD 0.53, as compared with USD 690 for 100 mL of Allprotect™ Tissue Reagent and USD 40 for a 700 mL bottle of Absolut vodka.

While the present study only involved the use of bovine flesh as a proxy for human soft tissue samples, we do not expect the presence of bone in a toe to pose any challenge. Previous literature of similar comparative studies with bone samples has reported that salt is an effective preservation medium. De Arellano Sánchez et al. compared the preservation of porcine bone samples using salt, 99% ethanol–EDTA mixture, and TENT (Tris-EDTA-NaCl-Triton X100 (Thermo Fisher Scientific, Waltham, MA, USA)) buffer at both room temperature and at 35 °C for up to 30 days, and reported that the porcine bone samples preserved in salt had yielded significantly higher DNA compared with the two other chemical preservatives [9]. Connell et al. also tested the efficacy of solid salt in preserving both human muscle and bone samples for seven days. Full profiles were obtained from 100% of muscle and 83% of bone samples [8]. We, therefore, believe that in our proposed approach, the tissue sample would be well coated in a substantial excess of salt, which would serve to rapidly dehydrate and preserve the entire sample at ambient temperature for post-incident DNA-based identification.

In conclusion, the present study has demonstrated the feasibility of two (salt and vodka) readily available ambient temperature tissue preservation options alongside chemical preservatives and freezing. While all four preservation methods yielded DNA that could still be STR-PCR amplified even after 24 months of storage, salt would be the recommended approach when tangible (e.g., recovery of high-molecular-weight DNA, low cost) and intangible (e.g., ease of scaling up and application) factors are taken into consideration.

## Data Availability

The raw data supporting the conclusions of this article will be made available by the authors upon request.

## Appendix A

**Table A1:** Summary of the results of this study and the mean input DNA amount for PCR-STR. DNA yield values provided are means of six readings from two cows, with standard deviations, while the input amount of DNA for PCR-STR was derived from the mean DNA yield divided by 56 (final DNA elution volume). DNA yield and input amount of DNA values are rounded to the nearest whole number. Percentage allele recovery provides an indication of the completeness of the bovine DNA profile recovered from the fresh control and the preserved samples. Locus SPS115 was excluded from analyses.

	Fresh	7 Days	1 Month	4 Months	12 Months	16 Months	24 Months
**Mean DNA Yield (ng)**							
Fresh control	1922 ± 331						
Salt		1892 ± 547	1983 ± 264	1850 ± 316	1917 ± 234	2000 ± 192	2750 ± 453
Allprotect™		2446 ± 426	2550 ± 491	2267 ± 662	1392 ± 501	1075 ± 272	1579 ± 695
Vodka		3283 ± 609	2792 ± 357	1533 ± 434	1200 ± 182	626 ± 407	686 ± 440
Freezing		2767 ± 489	2442 ± 540	1758 ± 357	1708 ± 267	171 ± 172	58 ± 54
**Mean Input Amount of DNA Used for PCR-STR (ng)**							
Fresh Control	34						
Salt		34	35	33	34	36	49
Allprotect™		44	46	40	25	19	28
Vodka		59	50	27	21	11	12
Freezing		49	44	31	31	3	1
**Percentage Allele Recovery (%)**							
Fresh Control	100						
Salt		100	100	100	100	100	100
Allprotect™		100	100	100	100	100	100
Vodka		100	100	100	100	100	100
Freezing		100	100	100	100	100	97

**Table A2:** Brown–Forsythe test results. The variance of the six readings at each time-point for each method of preservation was compared to the variance of twelve readings from unpreserved samples.

	Brown–Forsythe Test Statistic	*p*-Value
Salt	0.994	0.442
Allprotect™	0.699	0.652
Vodka	1.511	0.199
Freezing	2.931	0.018

**Table A3:** Shapiro–Wilk test results on DNA yield of samples preserved using each of the four methods. The number of data points for each method was 36 (6 readings across 6 time-points).

	Shapiro–Wilk Test Statistic	*p*-Value
Salt	0.937	0.040
Allprotect™	0.972	0.494
Vodka	0.940	0.046
Freezing	0.901	0.004
